# Validation of 24-hour ambulatory gait assessment in Parkinson's disease with simultaneous video observation

**DOI:** 10.1186/1475-925X-10-82

**Published:** 2011-09-21

**Authors:** Steven T Moore, Valentina Dilda, Bandar Hakim, Hamish G MacDougall

**Affiliations:** 1 Human Aerospace Laboratory, Department of Neurology, Mount Sinai School of Medicine, New York NY 10029, USA; 2 Robert and John M. Bendheim Parkinson and Movement Disorders Center, Department of Neurology, Mount Sinai School of Medicine, New York NY 10029, USA; 3 School of Psychology, University of Sydney, Sydney, Australia

**Keywords:** accelerometry, stride length, motor fluctuations, levodopa, Parkinsonian

## Abstract

**Background:**

Parkinson's disease (PD) is a neurodegenerative disorder resulting in motor disturbances that can impact normal gait. Although PD initially responds well to pharmacological treatment, as the disease progresses efficacy often fluctuates over the course of the day, and clinical management would benefit from long-term objective measures of gait. We have previously described a small device worn on the shank that uses acceleration and angular velocity sensors to calculate stride length and identify freezing of gait in PD patients. In this study we extend validation of the gait monitor to 24-h using simultaneous video observation of PD patients.

**Methods:**

A sleep laboratory was adapted to perform 24-hr video monitoring of patients while wearing the device. Continuous video monitoring of a sleep lab, hallway, kitchen and conference room was performed using a 4-camera security system and recorded to hard disk. Subjects (3) wore the gait monitor on the left shank (just above the ankle) for a 24-h period beginning around 5 pm in the evening. Accuracy of stride length measures were assessed at the beginning and end of the 24-h epoch. Two independent observers rated the video logs to identify when subjects were walking or lying down.

**Results:**

The mean error in stride length at the start of recording was 0.05 m (SD 0) and at the conclusion of the 24 h epoch was 0.06 m (SD 0.026). There was full agreement between observer coding of the video logs and the output from the gait monitor software; that is, for every video observation of the subject walking there was a corresponding pulse in the monitor data that indicated gait.

**Conclusions:**

The accuracy of ambulatory stride length measurement was maintained over the 24-h period, and there was 100% agreement between the autonomous detection of locomotion by the gait monitor and video observation.

## 1. Introduction

Parkinson's disease (PD) is a neurodegenerative disorder that results from a progressive loss of dopaminergic neurons, predominantly within the substantia nigra. One of the disabling clinical manifestations of PD is locomotor dysfunction; shortened stride length, increased variability of stride, reduced walking speed, and freezing of gait (a transient block of movement, particularly when initiating gait, turning, or negotiating an obstacle). Treatment of PD to a large extent focuses on replacing depleted dopamine at the striatum to improve locomotor function and maintain mobility for as long as possible [1]. While dopamine replacement therapy (most commonly with the dopamine precursor levodopa) is initially effective, most patients develop motor fluctuations after 3 years of treatment [2]. 'On' states, in which the motor symptoms of PD are effectively treated, may become shorter ('wearing off'), and periods of stiffness and difficulty of movement ('off' states) may occur unpredictably and more frequently [3].

Evaluation of long term medication response in PD usually takes the form of a patient diary, where the Parkinsonian state is noted as 'on', 'off' or 'on with dyskinesias (levodopa-induced involuntary movements)' [4,5], but these subjective records can be unreliable [6]. The Unified Parkinson's Disease Rating Scale (UPDRS) [7] is commonly used in research studies (and to a lesser extent in the clinic) to intermittently assess PD symptoms. Despite widespread acceptance (70% of PD clinical trials utilized the UPDRS from 1994-1998 [8]) it is a relatively blunt instrument; analysis of gait is limited to assigning a single value between 0 (normal) and 4 (unable to walk, even with assistance) from brief clinical observation. Motor function in PD patients can fluctuate markedly over the course of a day, and an objective long-term measure of gait may provide more effective titration of dopaminergic medication, better comparative treatment evaluations, and possibly a more accurate diagnosis of PD when administering the levodopa challenge test [9].

Wrist [10,11] and belt [12] mounted accelerometers have been used for long-term monitoring of gross motor function in PD, but this approach is unable to determine 'off' from 'on' states. A more 'brute force' approach (six tri-axial accelerometers; mounted on both upper arms, both upper legs, the sternum and one wrist) has been used to distinguish between 'on' and 'off' stages in PD [13], but the complexity of multi-segment accelerometry limits long-term use in the community. We have previously described a device for ambulatory assessment of gait using an array of linear and angular sensors (multi-axis accelerometer and gyroscope) placed on the lateral shank just above the ankle [14], which determines the length of every stride taken. In Parkinson's patients, the gait monitor has demonstrated sensitivity to motor fluctuations (rapid changes in stride length) following levodopa administration over short epochs (up to 3 h) [15,16] and can reliably detect episodes of freezing of gait from the frequency characteristics of vertical leg acceleration [17]. As motor fluctuations in PD occur over the entire day, the aim of the current study was to validate ambulatory monitoring of gait in PD patients over a 24-h epoch by a direct comparison with simultaneous video observation.

## 2. Methods

### 2.1 Participants

This study was approved by the Institutional Review Boards at Mount Sinai School of Medicine and IM Systems Inc. (Baltimore, MD); informed written consent was obtained from all participants. Stride length measures from the gait monitor were validated while walking along a 30-m hallway in healthy control subjects (N = 9; 5 males and 4 females; mean age 55 years (SD 2)), and PD patients (Table 1) prescribed oral levodopa (N = 4; 3 female and 1 male; 71 years (SD 6.4)). Three of these PD patients (2 female and 1 male; 73 years (SD 6.4)) participated in 24-h assessments of the gait monitor in a sleep laboratory, and during activities of daily living in the community.

**Table 1:** Patient Characteristics

Subject ID	Gender	Age (yr)	Disease Duration (yr)	Levodopa Dose Equivalency (mg)
006	F	66	12	600
007*	F	77	2	750
011*	M	76	6	700
012*	F	65	12	665

Characteristics of the four Parkinson's disease patients at the time of the study. All four subjects performed the stride length validation. Three patients (denoted by '*' adjacent to subject ID) participated in the 24-h video validation.

### 2.2 Gait monitor

A prototype gait monitor was developed by IM Systems based on a design described previously [14]. The sensor array consisted of a dual axis gyroscope (InvenSense IDG-300) and triaxial accelerometer (Freescale Semiconductor MMA7260QT). A 12 bit A/D converter sampled linear and angular leg movement at a rate of 100 Hz and saved the digital data on an SD flash memory card. An onboard 8-bit microcontroller was programmed to communicate with the PC software and, in conjunction with a real time clock, manage the start and stop of the data logging. This allowed the implementation of advanced features such as delayed recording, and accurate start and stop timing. The entire device, including a rechargeable lithium ion battery, was housed in a plastic enclosure (66 mm × 80 mm × 28 mm) and weighed 130 grams. Locomotor activity was identified by the appearance of power in the vertical acceleration trace in a narrow band centered on 2 Hz [18]; periods when the subject was lying down were determined from the change in baseline vertical acceleration from 9.8 m/s^2 ^to close to zero. Stride length was calculated from vertical acceleration and pitch angular velocity of the leg [14].

### 2.3 Stride length accuracy

Subjects (9 controls and 4 PD patients) were fitted with a chalk 'spur' attached to the rear of the left shoe, and asked to walk along a 30-m hallway while varying the length of their steps. The gait monitor was also worn on the left leg. After testing, the length of each stride was determined by measurement of the distance between successive chalk marks on the floor, and compared to the stride length determined by the gait monitor [14].

### 2.4 24-h ambulatory gait monitoring

The sleep laboratory at IM Systems was adapted to perform 24-hr video monitoring of PD patients while wearing the gait monitor. The sleep lab is a one-bed research facility with an armchair, TV and DVD player. Restroom/shower and kitchen facilities were adjacent. In addition, a 30-m hallway and conference room were available for patient use. Continuous video monitoring of the sleep lab, hallway, kitchen and conference room was performed using an integrated security system (VSS40DM 4-Camera DVR System, Supercircuits Inc, TX) and recorded to hard disk. The three PD patients participating in the 24-h study arrived at IM Systems in the early evening (around 5:00 pm). Following instrumentation, the 24 h period began with an accuracy validation along the 30-m hallway (see section 2.3 above), with the subject wearing the chalk 'spur' to allow a comparison between actual and measured stride length. Subjects stayed within IM Systems' premises over the 24-h epoch, and performed a final accuracy test prior to ending participation in the study the next evening.

Two independent observers (who were not privy to the gait monitor data) viewed the 24-h digital video records of the 3 PD subjects and independently coded gait activity (onset and cessation of locomotion) and when the subject was lying down, with a resolution of 1 s. If the subject walked into an area outside of the view of the surveillance cameras (the restroom or other blind spots), or walked into view from an unmonitored area, the gait activity was coded as a partial observation. After coding, the results from the two observers were compared and found to be almost identical; any instances of disagreement (a missed episode of locomotion, for example) were reconciled by both observers reviewing the period of video in question. The leg movement data from the gait monitor was processed to determine stride length and the onset and cessation of locomotor activity [14], and was synchronized with the video observation data (within 1 s) by alignment of the point at which the logger was activated by a button press (captured on video). In addition, the three PD patients wore the gait device for 24-h during normal daily activities in the community. Participants were requested to maintain a log of activities and when Parkinson's-related medications were taken.

## Results and Discussion

**Figure 1:**
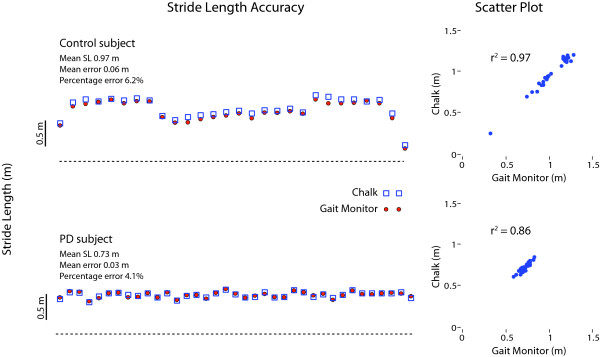
**Comparison of actual stride length (blue squares) and calculated stride length from the gait monitor (red circles) from a healthy control (upper left plot) and a PD subject (lower left plot)**. Subjects walked along a 30 m hallway wearing a chalk 'spur' to indicate heel strike. Scatter plots on the right hand side demonstrate the linear relationship between actual and measured stride length.

**Figure 2:**
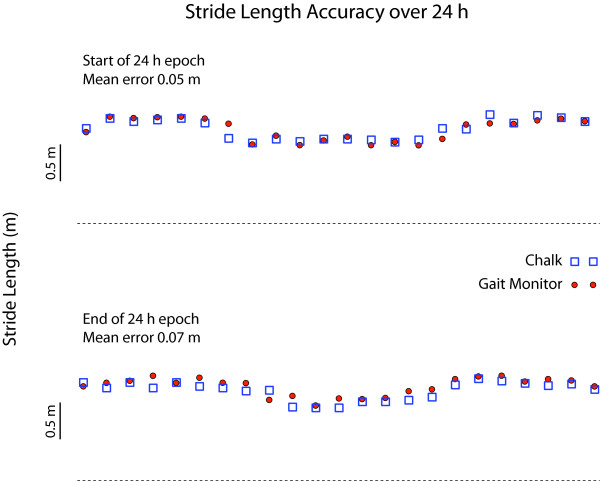
**Stride length from a PD patient while walking along a 30-m hallway as determined by chalk markings on the floor (blue squares) and the gait monitor (red circles)**. The top trace was acquired at the start of testing and the lower trace was acquired following 24-h of continuous gait monitoring. Accuracy in stride length measurement was maintained while wearing the gait monitor over the 24-h epoch.

**Figure 3:**
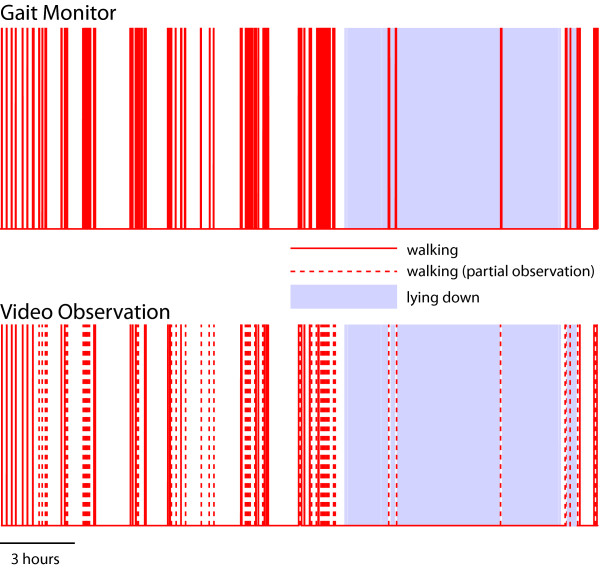
**Comparison of detection of locomotion and lying down in a PD patient by the gait monitor (top plot) and from video observation (lower plot) over 24 h**. A solid bar indicates that the subject was walking as determined by the gait monitor (upper plot) or video observation (lower plot). A partial observation (dashed line-lower trace only) indicates that the subject was out of view for a portion of the gait event. Shaded regions indicate periods when the subject was lying down. There was full agreement between the gait monitor results and independent video observation.

Mean stride length error while walking along a 30-m hallway was 0.064 m (SD 0.013; range 0.03-0.08 m) for the control group (N = 9) and 0.045 m (SD 0.024; range 0.02-0.07 m) for the four PD patients (Figure 1). Stride length accuracy was maintained over the 24-h recording epoch (Figure 2); the mean error at the start of recording in the subset of PD patients (N = 3) participating in this part of the study was 0.05 m (SD 0, range 0.05-0.05 m) and at the conclusion of the 24-h period was 0.06 m (SD 0.026; range 0.03-0.08 m). There was 100% agreement between identification of locomotor activity and lying down by the gait monitor and from video observation for all 3 PD subjects. For every period of walking noted by the device there was an accompanying period of locomotion coded in the video observations (Figure 3). This also held true for identification of when the subject was lying down.

**Figure 4:**
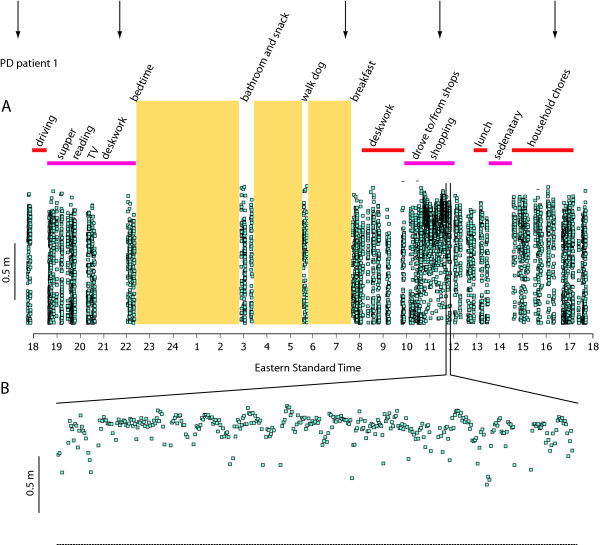
**Ambulatory gait monitoring over 24-h of activities of daily living**. (A) Stride length data (blue squares) and periods of lying down (shaded boxes) determined by the gait monitor over 24-h of community living in a subject with PD. The activities were reported by the subject in a simple diary. The vertical arrows indicate when PD related medications were self-administered. (B) Detail of 10-min of stride length while shopping.

**Figure 5:**
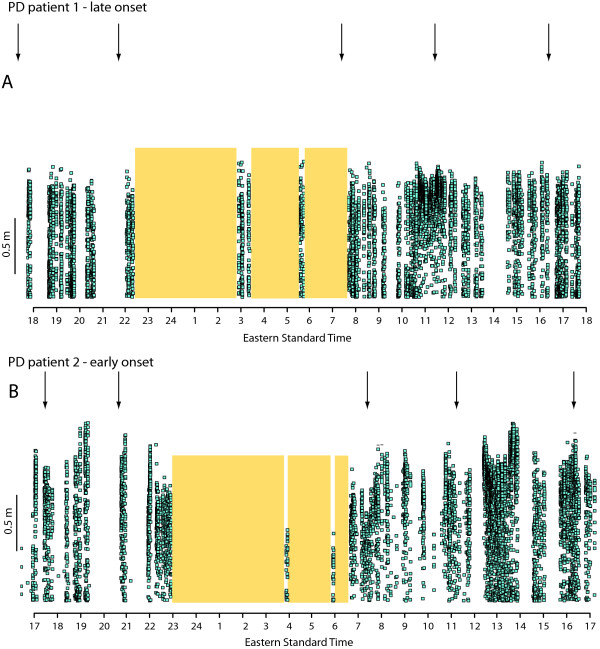
**Stride length data (blue squares) and periods of lying down (shaded boxes) during 24-h ambulatory activity in the community**. Comparison between the patient shown in Figure 4 (A) with a late onset and short disease duration (2 years), exhibiting a consistent stride length over the day, with (B) a PD patient with a longer disease duration (12 years) and relatively early onset (53 years). Note the fluctuations in stride length over the 24-h epoch in the latter patient.

The three PD patients enrolled in the 24-h in-house study also wore the device over an entire day of community living (Figure 4), keeping a simple written log of activities. No subjects reported any discomfort or interference with activities of daily living or sleep. A comparison of data from two PD patients over 24-h provides an indication of the potential application of the gait monitor (Figure 5). PD patient 1 (Figure 5A) was recently diagnosed (2 years ago) at the age of 75 years, whereas PD patient 2 (Figure 5B) was diagnosed relatively young (53 years) with a disease duration of 12 years. Both patients self-administered levodopa 5 times during the 24-h epoch (Figure 5A, B; arrows), but it is clear that PD patient 1 (Figure 5A) exhibited a more consistent stride length over the measurement period than patient 2.

The results of this study demonstrate the viability of ambulatory gait monitoring using an integrated sensor array/data logger placed on the lateral shank over periods of up to 24-h. Stride length accuracy was maintained over 24-h of continuous gait monitoring and there was full agreement between autonomous detection of locomotion (and periods of lying down) and video observation. Participants reported no discomfort or inconvenience wearing the device for extended periods, whether in the laboratory or during community activities. Data from 24-h community monitoring showed differences in motor fluctuations consistent with patient history; stride length was consistent over the day in a patient with late onset, short duration PD, whereas a patient with a younger onset and longer duration exhibited considerable fluctuations in stride length that appeared temporally linked to levodopa administration.

## Conclusions

The accuracy of ambulatory stride length measurement in PD patients was maintained over a period of 24-h. There was 100% agreement between the autonomous detection of locomotion and periods of lying down from shank-mounted accelerometer output and video observation by two independent raters. Long-term monitoring of gait in PD provides an objective dynamic assessment of motor fluctuations and may prove a useful adjunct in the management of Parkinson's disease and other movement disorders

## Competing interests

The authors declare that they have no competing interests.

## Authors' contributions

HGM designed and constructed the laboratory prototype gait monitor (which formed the basis for the IM Systems prototype); STM designed and implemented algorithms and software for processing of leg movement data to obtain stride length and activity, and wrote the manuscript; VD and BH performed the coding of patient activity from 24-h video recordings. All authors read and approved the final manuscript.
